# Aberrant Expression of Cx43 Is Associated with the Peritoneal Metastasis of Gastric Cancer and Cx43-Mediated Gap Junction Enhances Gastric Cancer Cell Diapedesis from Peritoneal Mesothelium

**DOI:** 10.1371/journal.pone.0074527

**Published:** 2013-09-11

**Authors:** Bo Tang, Zhi-hong Peng, Pei-Wu Yu, Ge Yu, Feng Qian, Dong-zhu Zeng, Yong-liang Zhao, Yan Shi, Ying-xue Hao, Hua-xing Luo

**Affiliations:** 1 General Surgery Center of PLA, Southwest Hospital, the Third Military Medical University, Chongqing, China; 2 Institute of Digestive Diseases, Southwest Hospital, the Third Military Medical University, Chongqing, China; University Hospital Hamburg-Eppendorf, Germany

## Abstract

The process of peritoneal metastasis involves the diapedesis of intra-abdominal exfoliated gastric cancer cells through the mesothelial cell monolayers; however, the related molecular mechanisms for this process are still unclear. Heterocellular gap-junctional intercellular communication (GJIC) between gastric cancer cells and mesothelial cells may play an active role during diapedesis. In this study we detected the expression of connexin 43 (Cx43) in primary gastric cancer tissues, intra-abdominal exfoliated cancer cells, and matched metastatic peritoneal tissues. We found that the expression of Cx43 in primary gastric cancer tissues was significantly decreased; the intra-abdominal exfoliated cancer cells and matched metastatic peritoneal tissues exhibited increasing expression compared with primary gastric cancer tissues. BGC-823 and SGC-7901 human gastric cancer cells were engineered to express Cx43 or Cx43T154A (**a** mutant protein that only couples gap junctions but provides no intercellular communication) and were co-cultured with human peritoneal mesothelial cells (HPMCs). Heterocellular GJIC and diapedesis through HPMC monolayers on matrigel-coated coverslips were investigated. We found that BGC-823 and SGC-7901 gastric cancer cells expressing Cx43 formed functional heterocellular gap junctions with HPMC monolayers within one hour. **A** significant increase in diapedesis was observed in engineered Cx43-expressing cells compared with Cx43T154A and control group cells, which suggested that the observed upregulation of diapedesis in Cx43-expressing cells required heterocellular GJIC. Further study revealed that the gastric cancer cells transmigrated through the intercellular space between the mesothelial cells via a paracellular route. Our results suggest that the abnormal expression of Cx43 plays an essential role in peritoneal metastasis and that Cx43-mediated heterocellular GJIC between gastric cancer cells and mesothelial cells may be an important regulatory step during metastasis. Finally, we observed that the diapedesis of exfoliated gastric cancer cells through mesothelial barriers is a viable route of paracellular migration.

## Introduction

The incidence of gastric cancer is declining but remains a major cause of cancer-related death worldwide. The predominant features of gastric cancer cells include their invasive and metastatic capacities. Peritoneal metastasis is a critical feature for tumor progression in advanced gastric cancer [1]. During the process of peritoneal metastasis, gastric cancer cells interact intercellularly with multiple cell types, and the interaction of the intra-abdominal exfoliated gastric cancer cells and the peritoneal mesothelial cells is of particular importance. As continuous human peritoneal mesothelial cell (HPMC) monolayers act as a barrier against peritoneal metastasis, once the diapedesis of exfoliated gastric cancer cells through mesothelial cell monolayers occurs, the abundant blood supply in the matrix beneath the mesothelium will offer a comfortable environment for metastatic gastric cancer cells and promote their colonization. Therefore, diapedesis of exfoliated gastric cancer cells through the mesothelial cell monolayer is an essential step during peritoneal metastasis. The interaction between these cells is accompanied by intercellular communication (IC), which is predominantly mediated by cell-cell gap junctions [2]. Gap junctions are formed by plasma membrane connexins, each of which is comprised of six connexins (Cxs) [3]. To date, 20 different connexin isoforms have been identified in humans. Cx43 is a general isoform expressed in most epithelial tissues. Previous studies [4–6] indicated that Cx43 expression decreased during tumorigenesis and was therefore classified as a tumor suppressor. However, there is a growing body of evidence that connexins may be involved in the intravasation and extravasation of cancerous cells and play a positive role in the process of metastasis [7,8]. Whether the Cx43 mediated gap junction plays an important role in diapedesis of gastric cancer cells through the peritoneal mesothelial barrier remains unclear. To address the hypothesis we examined the expression of Cx43 in primary gastric cancer tissues, exfoliated gastric cancer cells, and peritoneal metastatic tissues. We constructed a Cx43-expressing vector and a Cx43T154A site mutation vector. Then, we used two human gastric cancer cell lines (BGC-823 and SGC-7901) that is GJIC deficient and does not express any known connexins [9]. Because mesothelial cells abundantly express Cx43, we engineered gastric cancer cells to express either wild-type Cx43 or a site-specific mutant to determine whether the potential of gastric cancer cells’ diapedesis through the mesothelium would change under both or either of these conditions. In this study we show that Cx43 expression upregulates tumor cell diapedesis via a GJIC-dependent mechanism.

## Materials and Methods

### 2.1: Reagents and Antibodies

Anti-connexin 43 polyclonal antibody (Zymed, San Diego, CA, USA), Matrigel (Becton-Dickenson, Bedford, MA, USA), 1, 1’-diocta-decyl-3, 3, 3’, 3’-tetramethylindocarbocyanine percholate (Dil; Molecular Probes, Eugene, OR, USA), Calcein-AM (Dojindo, Kumamoto, Japan), and FITC-conjugated phalloidin were purchased from Sigma (Sigma-Aldrich, St. Louis, MO, USA).

### 2.2: Tissue samples and Cytological Examination

All participants provided written informed consent (from their guardians where necessary). This study was conducted in accordance with the tenets of the Declaration of Helsinki and its amendments and was approved by the ethics committee of Southwest hospital, The Third Military Medical University. The study population consisted of 42 patients who were classified as stage IV according to the seventh edition of UICC TNM Classification of Malignant Tumors. Clinicopathologic variables are listed in Table 1. All of the patients enrolled in our study underwent palliative or exploratory surgery. Primary tumor and metastatic peritoneal tissues were collected before the end of surgery, and exfoliated gastric cancer cells were harvested by the centrifugation of ascites or peritoneal lavage fluid (if ascites did not exist). The nucleated cell layer was smeared onto a glass slide and stained with hematoxylin and eosin (HE). Two experienced cytopathologists performed the cytological evaluations. CEA was used as a biomarker to help identify adenocarcinoma cells by immunofluorescence with positive staining. Cases were excluded from the present study when the findings of both of the cytopathologists were not in agreement.

**Table 1:** Clinical and pathological characteristics of 42 cases of patients with gastric cancer.

**group**	**Number**	**percent (%**)
**Sex**		
male	27	64.0
female	15	36.0
**Age(years**)		
<50	17	40.4
≥50	25	59.6
**Tumor location**		
Antral	21	50.0
Body	12	28.6
Fundic	9	11.4
**Pathological type**		
Well differentiated	2	4.7
Moderately differentiated	4	9.5
Poorly differentiated	36	85.8
**Bormann typing**		
I	0	0
II	1	2.4
III	12	28.5
IV	29	69.1
**Ascites**		
Yes	15	35.7
No	27	64.3
**Surgical approach**		
palliative gastrectomy	24	57.1
exploratory+biopsy	18	42.9

### 2.3: Immunohistochemistry

Paraffin tissue samples of primary tumors and metastatic peritoneal tissues were serially sectioned, and HE staining confirmed the existence of gastric cancer. Consecutive sections were deparaffinized, dehydrated, and subjected to antigen retrieval in sequence. The sections were incubated with a rabbit polyclonal anti-Cx43 antibody for 24 hours at 4°C. Binding sites were visualized with 3, 3’-diamino-benzidine (DAB) in a 5-min reaction. The sections were counterstained with Mayer’s hematoxylin, dehydrated, cleared and mounted. Omission of the primary antibody was used as a negative control. Immunohistochemical staining was evaluated by assigning a score based on the extent and intensity of immunoreactivity. Staining extent was evaluated semiquantitatively in cytoplasm and membrane as negative (0, <5% cells stained), positive 1 + (6–25% cells stained), positive 2 + (26–50% cells stained) or positive 3 + (>50% cells stained). The staining intensity was scored as 0 (no staining), 1 (weak staining, yellow brown), 2 (moderate staining, yellow brown), or 3 (strong staining, brown). Immunostaining results were scored as the sum of the extent and intensity of immunoreactivity, considering a score ≥3 positive and a score <3 negative.

### 2.4: Immunofluorescence

Cx43 expression in intra-abdominal exfoliated gastric cancer cells was assessed using immunofluorescence. Forty-two cases were enrolled in our study. The cells smeared on glass coverslips were fixed with 4% paraformaldehyde and washed with PBS containing 0.5% BSA (rinsing buffer). The cells were then incubated for 24 hours at 4°C with an anti-Cx43 antibody (1:150). The coverslips were washed and incubated with FITC-conjugated goat anti-rabbit secondary antibody. The nuclei were stained using 10 µg/ml DAPI (Sigma-Aldrich, St. Louis, MO, USA). The coverslips were imaged using a fluorescence microscope (Siemens, Germany). The controls (primary antibodies were replaced by PBS) were negative for all experiments (data not shown).

### 2.5: Cell culture and Stable expression of Cx43 and Cx43 mutants

Nonmalignant human immortalized mesothelial cells, MeT-5A, were obtained from ATCC. These cells were maintained and propagated in Medium 199 containing 1.5 g/L sodium bicarbonate, 10% fetal bovine serum, 3.3 nM epidermal growth factor (EGF), 400 nM hydrocortisone, 870 nM zinc-free bovine insulin, 20 mM HEPES, and 3.87 µg/L selenious acid (H2SeO3).

The human gastric adenocarcinoma cell lines BGC-823 and SGC-7901 were obtained from the Shanghai Institute cell bank, Chinese Academy of Science. Cx43- and Cx43T154A-expressing vectors were engineered using lentiviral infection. PTA2-Cx43, which included a human Cx43-encoding sequence, was constructed in our laboratory. The Cx43T154A site mutation vector construction was accomplished using Overlap extension PCR, which encoded a protein that functioned as a gap junction without IC as previously described [10]. The cDNA of Cx43 and Cx43T154A was inserted into the lenti-GFP vector and transfected into the 293T packaging cell line. The lentiviral supernatant was collected after 48 h, filtered through a 0.45-µm filter and used to infect BGC-823 and SGC-7901 cells. The medium was replaced with RPMI1640 24 hours after infection, and the cells were cultured routinely. More than 90% of the tumor cells expressed Cx43 or Cx43T154A after three rounds of viral infection; the successfully infected cells were collected by flow cytometry.

### 2.6: Protein extraction and Western blot analysis

The engineered Cx43- and Cx43T154A-expressing BGC-823 and SGC-7901 gastric cancer cells were lysed. Protein (50 µg) from each cell type was separated in a 12% SDS-polyacrylamide gel and transferred to nitrocellulose membranes. Nitrocellulose membranes were blocked with 5% nonfat dry milk and were then probed for the Cx43 protein using a polyclonal anti-Cx43 antibody (1:500). Immunoblots were probed with the appropriate horseradish peroxidase-conjugated secondary antibodies (Sigma-Aldrich, St. Louis, MO, USA) at a 1:10,000 dilution. The proteins were visualized using a Supersignal West Pico Chemiluminescent substrate (Pierce Chemical Co.) and immunoblots were exposed to Amersham Hyperfilm (Amersham Biosciences, Piscataway, NJ, USA) for 30 s.

### 2.7: Tumor Cell/Mesothelial Cell Adhesion Assay

Mesothelial cells were grown until monolayer confluence on glass coverslips in 24-well plates. The engineered Cx43- and Cx43T154A-expressing gastric cancer cells were labeled with a 10 µg/ml Dil solution for 15 min at 37°C. Dil is a commonly used fluorescence probe that shows an orange-red fluorescence in membranes and was used in our study to label gastric cancer cells. These labeled cells were then added to the mesothelial cell monolayers and incubated for 1 h at 37°C. Nonadherent cells were removed by washing, and the cells that had adhered to the mesothelial cell monolayers were fixed with 4% paraformaldehyde. The culture slides were mounted in Mowiol (Calbiochem) and examined using fluorescence microscopy (Leica MPS 60).

### 2.8: Tumor Cell/Mesothelial Cell GJIC assay

The engineered Cx43- and Cx43T154A-expressing BGC-823 and SGC-7901 gastric cancer cells were labeled for 15 minutes at 37°C with 2 ml of Opti-MEM containing 10 µg/ml calcein-AM and 10 µg/ml Dil. Calcein-AM is a fluorescent substrate that is cleaved in viable cells to a membrane-impermeable form that may pass through functional gap junctions but not other plasma membrane channels. Mesothelial cell monolayers were pretreated for 4 h with 150µM carbenoxolone (CBX) to block homotypic GJIC and were then treated for 15 minutes with 10 µg/ml calcein-AM (Molecular Probes) in an Opti-MEM solution. The well prepared labeled gastric cancer cells were added to the MeT-5A monolayers on the Matrigel, and the heterotypic GJIC between the gastric cancer cells and the mesothelial cells was detected. Fluorescence recovery in bleached cells will only occur if dye passes through the gap junctions of adjoining unbleached gastric cancer cells. Individual mesothelial cells adjacent to gastric cancer cells were bleached for approximately 30 s using a Zeiss laser scanning confocal microscope and an argon laser at 33% power for each experimental condition. Single mesothelial cells that could not establish GJIC were chosen to undergo blenching as controls. Untreated cells were used as background modifying cells. The mean fluorescence intensity of bleached/unbleached cells was measured over time using Scion imaging software, and the fluorescence recovery rate was calculated as well (Kt = (Ib-Ib0)/ Iu×100%, Kt: fluorescence recovery rate at time t; Ib: the fluorescence intensity in the bleached cell at time t; Ib0: the fluorescence intensity in the bleached cell at time t = 0; Iu: fluorescence intensity in the adjacent unbleached cell at time t) [11].

### 2.9: Transmesothelial migration assay

The transmesothelial migration assay was performed, as described previously [12]. Briefly, 1×10^5^ MeT-5A mesothelial cells were seeded onto 12-mm glass coverslips that were coated with Matrigel (Becton-Dickenson, USA). Monolayers reached confluence as determined using light microscopy. Coverslips were transferred to a 24-well plate and were cultured for 48 h in EGM. Gastric cancer cells were divided by empty vector group, Cx43-expressing group, Cx43T154A-expressing group and CBX pretreated group (Cx43-expressing gastric cancer cells were pretreated for 4 h with 150µM carbenoxolone (CBX) to block homotypic GJIC) and labeled with Dil. Approximately 1×10^5^ cells were added to mesothelial cell monolayers at a ratio of 1:10 (tumor cell: mesothelial cell). The cells were co-cultured for different times (1, 4, or 7 h) prior to live observation or fixation and immunostaining. Blinded investigators quantified diapedesis using LSM, as described previously [7]. Briefly, co-cultures were fixed and stained for F-actin. Diapedesis was quantified as the number of tumor cells that were in contact with the mesothelium and classified into three stages according to their position relative to the mesothelium: 1) rounded - tumor cells with a spherical shape on the apical surface of the mesothelium; 2) migrating - tumor cells had penetrated through the mesothelial cell monolayers at mesothelial cell junctions with a portion of the cell body above the mesothelium and a portion of the cell body spread on the Matrigel beneath the mesothelial cell F-actin stress fibers; and 3) underneath - the entire tumor cell body was below the plane of the mesothelial cell stress fibers. Migrating and underneath cells were classified as transmigrating. Approximately 100 mesothelium-adherent cells were recorded and counted per coverslip, and all experiments were repeated three times with triplicate coverslips.

### 2.10: Statistical analysis

All statistical analysis was performed using the SPSS software (version 13.0). Probabilities of P < 0.05 were considered statistically significant.

## Results

### 3.1: Cx43 expression in primary tumors and matched metastatic peritoneal tissues

**Figure 1:**
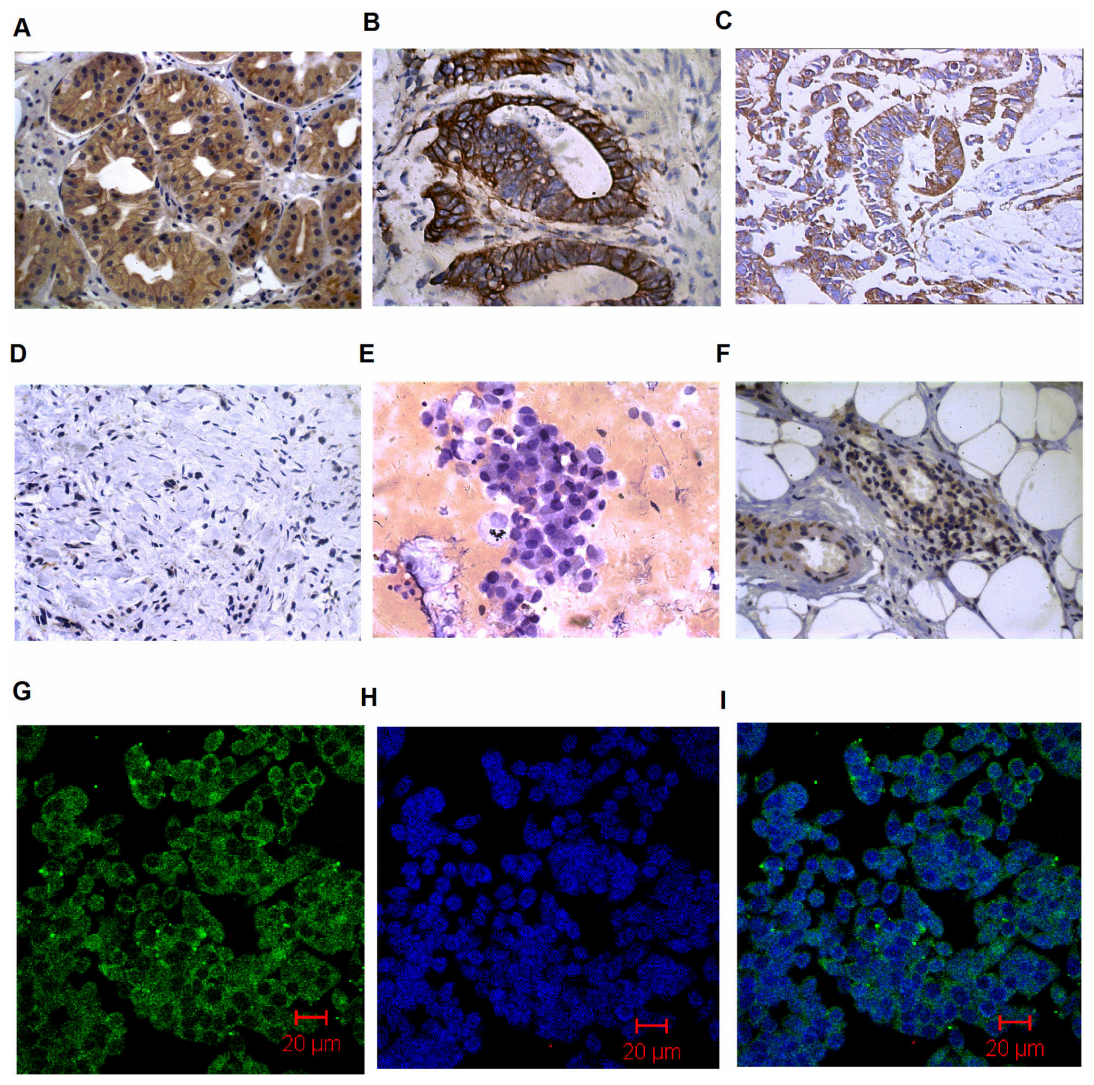
Cx43 expression in normal gastric tissue specimens, primary gastric carcinoma tissues, matched metastatic peritoneal tissues and intra-abdominal exfoliated gastric cancer cells. (A) Cx43 was expressed in normal epithelia with typical membranous and cytoplasmic staining. (B) Cx43 expression in well-differentiated gastric cancer tissues, cancer cells showed a positive membrane and cytoplasmic staining pattern. (C) Cx43 showed a major positive staining in cytoplasm in moderately differentiated gastric cancer tissues (D) Cx43 expression in poorly differentiated gastric cancer tissues, tumour cells show a negative staining pattern. (E) Cx43 expression in metastatic peritoneal tissues showed a positive staining pattern. (F) Intra-abdominal exfoliated gastric cancer cells were determined by Hematoxylin and eosin (H and E) staining. (G, H, I) The expression of Cx43 in intra-abdominal exfoliated gastric cancer cells was accessed by immunofluorescence. Original magnification: (A-F, ×400).

Forty-two normal epithelia (100%) expressed Cx43 with typical membranous and cytoplasmic staining (Figure 1A). Eleven of 42 primary tumors (26.2%) were positive for Cx43, which was significantly decreased compared to adjacent normal epithelia (p<0.05). Cx43 expression was heterogeneous in different tissue samples: in well differentiated gastric cancer tissues, Cx43 expression showed a major mixed (cytoplasmic and membranous) staining (Figure 1B); moderately differentiated gastric cancer tissues had only cytoplasmic Cx43 expression (Figure 1C); and Cx43 was scarcely expressed in poorly differentiated gastric cancer tissues (Figure 1D). In contrast, Cx43 expression was significantly increased in 29 of the 42 metastatic peritoneal tissues (69.0%) and exhibited typical cytoplasmic and membranous staining (p<0.05) (Figure 1E) (Table 2).

**Table 2:** Immunohistochemistry results for Connexin 43 expression in primary gastric cancer tissues, adjacent normal gastric tissues and peritoneal metastatic tissues (N=42).

Connexin 43	Primary gastric cancer (cases) (%)	Adjacent normal tissue (cases) (%)	Metastatic peritoneal tissue (cases) (%)
Negative(score <3)	31 (73.8%)	0(0)	13 (31.0%)
Positive (score≥3)	11 (26.2%)*	42 (100%)	29 (69.0%)†

* Compared to adjacent normal tissues, the expression of connexin 43 decreased significantly (p<0.05); †Compared to primary gastric cancer tissues, the expression of connexin 43 in metastatic peritoneal tissue increased significantly (p<0.05).

### 3.2: Cx43 expression in intra-abdominal exfoliated gastric cancer cells

Most intra-abdominal exfoliated gastric cancer cells exist as multicellular spheroids (Figure 1F). Positive Cx43 immunostaining was observed in 35 of the 42 cases of intra-abdominal exfoliated cancer cells with mixed cytoplasmic and membranous staining, and the positive rate is 83.3% (Figure 1 G, H, and I).

### 3.3: Expression of exogenous Cx43 in BGC-823 and SGC-7901 gastric cancer cells

**Figure 2:**
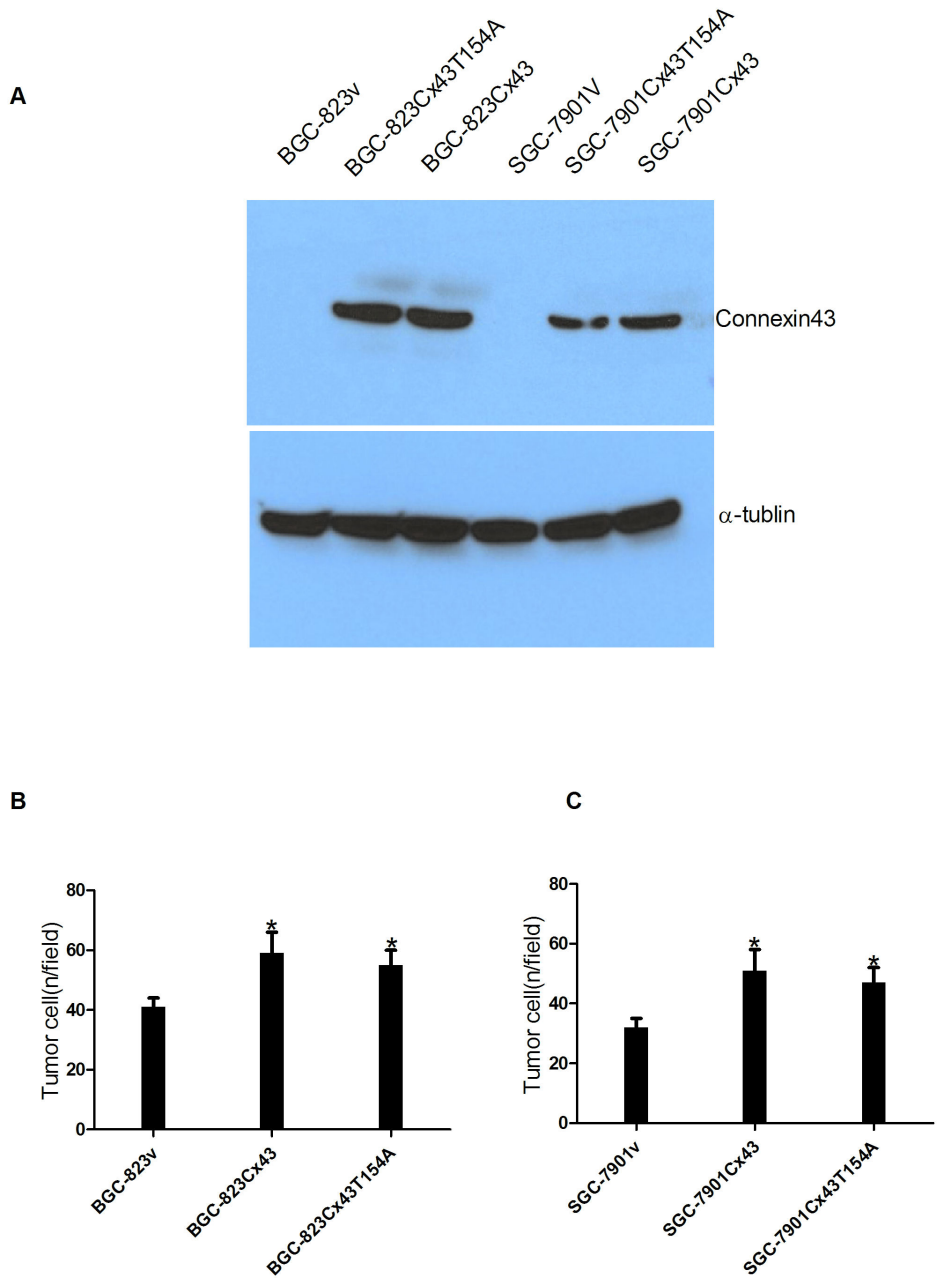
Effects of Cx43 on in vitro adhesion of BGC-823 and SGC-7901gastric cancer cells to mesothelial cells after transfection. (A) Cx43 and Cx43T154A expression of BGC-823 and SGC-7901 gastric cancer cells after transfection. (B, C) Effects of Cx43 on in vitro adhesion of BGC-823 and SGC-7901 gastric cancer cells to mesothelial cells. Adherent tumor cells were counted (magnification: ×200), the results showed that adhesion of cells transfected with Cx43 or Cx43T154A was significantly increased compared to that of cells transfected with empty vector (*P<0.05). Data are presented as the mean± SEM.

Gastric cancer cells were engineered to express functional Cx43, Cx43T154A and an empty lentiviral vector as a control to investigate the effects of Cx43 expression on the biological behavior of these cells. Cx43 expression was absent in the empty vector group, but the other two engineered groups were detected to express either Cx43 or Cx43T154A (Figure 2A).

### 3.4: Adhesion Properties of Gastric Cancer Cells to Mesothelial Cells

The effect of Cx43 on the adhesion of gastric cancer cells to the mesothelial cells was also investigated. Engineered gastric cancer cells expressing either wild-type Cx43 or Cx43T154A exhibited a significant increase in adhesion to mesothelial cells compared with empty vector group (P<0.05). No significant differences in adhesion ability were observed between Cx43- and Cx43T154A-expressing groups (P>0.05), suggesting that Cx43 promotes gastric cancer cell adhesion, which is independent of GJIC (Figure 2B).

**Figure 3:**
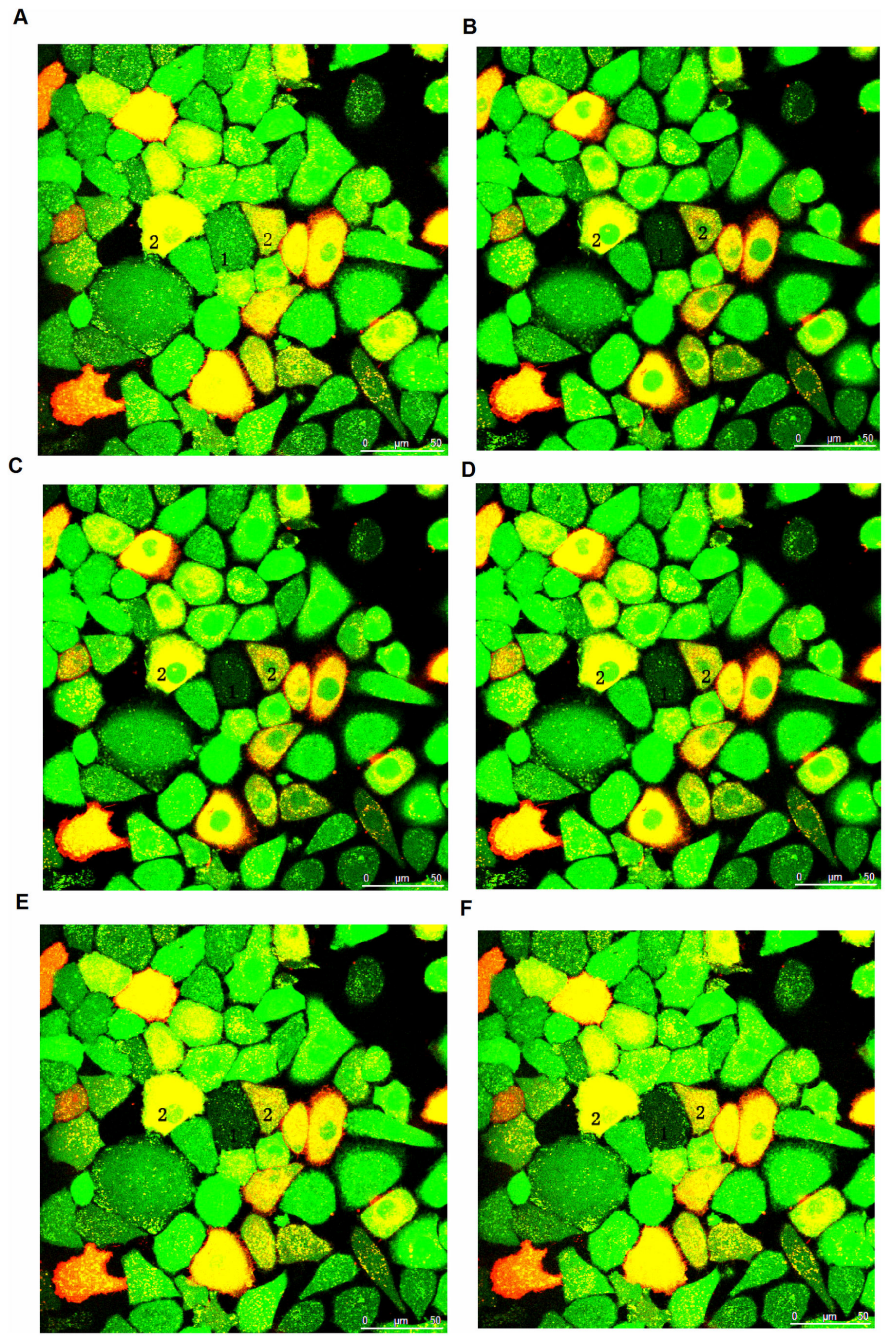
Fluorescence recovery images after photobleaching. 1.photobleaching mesothelial cells; 2.adjacent gastric cancer cells. A, before bleaching; B, C, D, E, F representing images in 0, 1, 2, 3, 4min after photobleaching, respectively.

**Figure 4:**
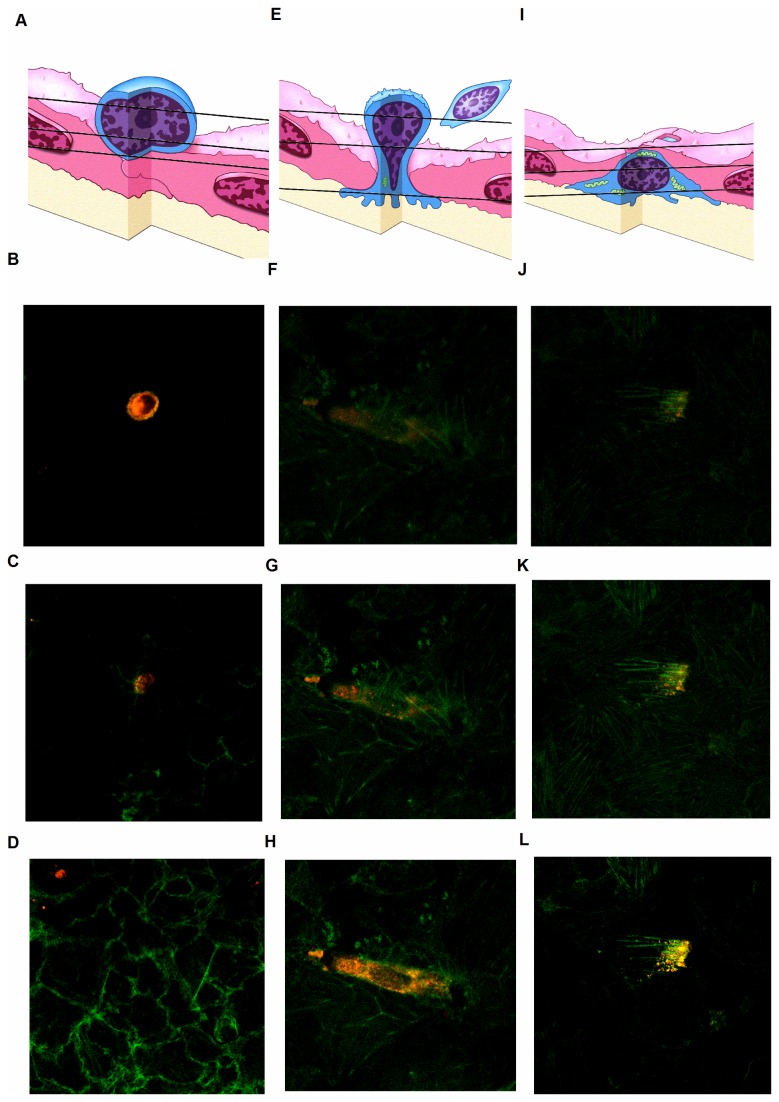
Tumor cell diapedesis through mesothelial cell monolayers. Optical sections obtained by laser scanning confocal microscopy at the focal levels indicated in (A, E, I) identified three major stages of diapedesis: round on top of (A–D), migrating through (E–H), or located underneath (I–L) the mesothelium. (B) Gastric cancer cells with a round shape had filopodial extensions presented in the cytoplasm (arrows). (H) Gastric cancer cells located in mesothelial cell-cell junctions had long spindle and thick bundles of F-actin presented close to cytoplasm (arrows). (K, L) Those that completed diapedesis showed prominent stress fibers distributed loosely in cytoplasm (arrows).

### 3.5: Engineered Cx43-expressing BGC-823 and SGC-7901 cells form functional gap junction channels with mesothelial cell monolayers

We co-cultured engineered Cx43- and Cx43T154A-expressing gastric cancer cells with mesothelial cell monolayers to examine the ability of Cx43-expressing cells to form gap junctions and establish GJIC with mesothelial cells. The results show that gastric cancer cells in either the empty vector group or the Cx43T154A group failed to establish GJIC with adjacent peritoneal mesothelial cells after laser bleaching, whereas the Cx43-expressing group was able to establish GJIC with peritoneal mesothelial cells successfully. The intracellular fluorescence intensity was detected before bleaching, at the instant of bleaching, and either 1 min, 2 min, 3 min or 4 min after bleaching. The results show that, in Cx43-expressing group, the intracellular fluorescence intensity of bleached mesothelial cell decreased rapidly after bleaching. Fluorescence intensity then gradually recovered due to dye passage through gap junctions with adjoining unbleached gastric cancer cells, and the mean fluorescence recovery rate was 35.6±0.9% and 39.4±0.8% at 4 min after blenching in BGC-823 and SGC-7901 cells, respectively, which was significantly higher than that in Cx43T154A and empty vetor group (p<0.05) (Figure 3, Table 3).

**Table 3:** Comparison of the mean fluorescence recovery rates after bleaching in BGC-823 and SGC-7901 gastric cancer cells (fluorescence intensity, *x*±s).

Group	Mean fluorescence recovery rate(%,*x*±s) (t=4min)
BGC-823Cx43	35.6±0.9*
BGC-823Cx43T154A	12.1±0.6
BGC-823v	13.4±0.7
SGC-7901Cx43	39.4±0.8†
SGC-7901Cx43T154A	15.3±0.4
SGC-7901v	14.7±0.6

* Compared to BGC-823v and BGC-823Cx43T154A, the mean fluorescence recovery rate in BGC-823Cx43 gastric cancer cells was significantly higher at time=4min (*p<0.05); †Compared to SGC-7901v and SGC-7901Cx43T154A, the mean fluorescence recovery rate in SGC-7901Cx43 group was significantly higher at time= 4min (*p<0.05).

### 3.6: Morphological evaluation of tumor cell diapedesis

We established an *in vitro* system to image the transmesothelial migration of gastric cancer cells to investigate the mechanism and route of diapedesis. This assay mimics aspects of the vessel wall and has been used previously to analyze leukocyte diapedesis [11]. Dil-labeled tumor cells were scored according to their morphology and location with respect to the mesothelium. We defined three distinct stages of migration using confocal microscopy: rounded (Figure 4A); migrating (Figure 4E); and underneath (Figure 4I). Tumor cells on top of the mesothelium prior to diapedesis exhibited a round or ovular shape, and this shape was maintained from the apical (Figure 4B) to the basal surface (Figure 4C, D). Migrating tumor cells passing between adjacent mesothelial cells changed shape from ovular to a long spindle with intracellular F-actin assembled into actin filament bundles (Figure 4F, G, H). The tumor cells that completed diapedesis were located completely underneath the mesothelium. The cell shape gradually changed from the long spindle to the rounder shape, and the intracellular F-actin filaments gradually extended and were a sparsely arranged between filaments (Figure 4J, K, L). Our study revealed that gastric cancer cells’ diapedesis occurred via a paracellular route.

### 3.7: Cx43-mediated GJIC enhanced gastric cancer cells diapedesis

**Figure 5:**
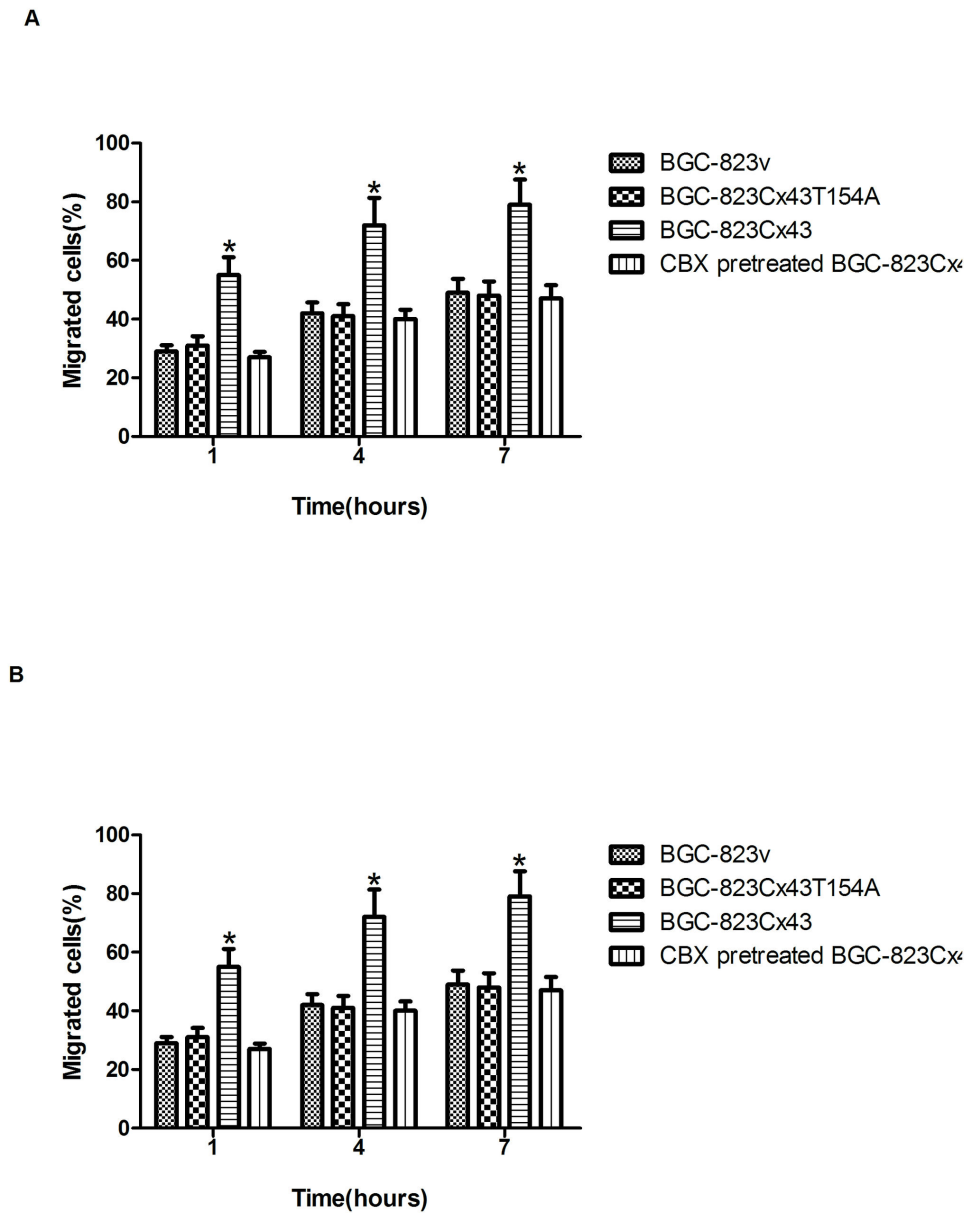
Connexin 43 mediated gap junctional intercellular communication enhances tumor cell diapedesis. Adherent cells in the process of diapedesis and cells that had completed diapedesis were scored together as having migrated cells successfully. (A) Percentage of adherent migrating cells in BGC-823 group cells(%), compared to BGC-823v (control), a significant increase in diapedesis of BGC-823Cx43 cells was observed at 1, 4 and 7 h (*p<0.05); (B) Percentage of adherent migrating cells in SGC-7901 group cells(%), compared to SGC-7901v (control), a significant increase in diapedesis of SGC-7901Cx43 cells was observed at 1, 4 and 7 h (*p<0.05).

We co-cultured Cx43-, Cx43T154A-expressing gasric cancer cells and CBX pretreated Cx43-expressing cells with mesothelial cells for 1, 4 and 7 h to compare their diapedesis efficiency [1,2]. These cells were scored using the described criteria for migration. The percentage of migrating Cx43T154A and CBX pretreated cells were not significantly different to empty vector group cells after 7 h of co-culture (p>0.05), but the diapedesis efficiency of Cx43-expressing group cells was significantly higher than other three groups (p<0.05) (Figure 5). These results suggest that the presence of the Cx43 protein in the tumor cell alone is not responsible for the observed increase in diapedesis, but that heterocellular GJIC between tumor cells and mesothelial cells is required to augment the efficiency of diapedesis for gastric cancer cells. These results suggested that Cx43 facilitates GJIC-dependent tumor cell diapedesis.

## Discussion

Gap junctions and connexin subunits are downregulated in various cancers [4,13,1,4]. However, recent reports indicate that connexins’ expression is not always maintained at decreased levels during tumor progression; conversely, they may be present during the later stages of carcinogenesis, increasing the migration capacity of invasive cells [2,15–11,7]. This re-emergence of connexins is clearly observed during the progression of human breast cancer, where Cx26- and Cx43-negative primary tumors developed Cx26- and Cx43-positive metastases in lymph nodes [18,1,9]. Similar results have also been obtained in mouse skin carcinogenesis; Cx26 expression is reduced during the early stages of carcinogenesis but is restored in metastatic lymph nodes [20]. These studies imply that connexins play different roles during the process of carcinogenesis. Our study was the first time to examine the expression of Cx43 during different stages of gastric cancer progression (primary gastric cancer tissues, intra-abdominal exfoliated gastric cancer cells and matched metastatic peritoneal tissues). The results show a significant decrease in Cx43 expression in primary gastric cancer tissues in comparison with the adjacent normal gastric tissues (p<0.05). In contrast, Cx43 expression in intra-abdominal exfoliated gastric cancer cells and metastatic peritoneal tissues was significantly increased. These results suggest that Cx43 most likely plays an important role in peritoneal metastasis.

To explore whether Cx43 expression has an effect on peritoneal metastasis, Cx43 was transfected into BGC-823 and SGC-7901 gastric cancer cells, and a Cx43T154A site mutation was constructed to determine the role of gap junctional communication in the process of peritoneal metastasis. Adhesion and diapedesis of tumor cells to the mesothelial cell monolayers are two critical steps for the formation of peritoneal metastasis. An *in vitro* cell adhesion assay revealed that Cx43- and Cx43T154A-expressing BGC-823 and SGC-7901 cells exhibited more efficient adhesion to the mesothelial cell monolayers than empty vector cells and that the adhesion efficiency of Cx43T154A-expressing tumor cells was similar to Cx43-expressing cells. These results suggest that Cx43 promotes the adhesion of gastric cancer cells to mesothelial cells, which is independent of the gap junction communication. With regard to its related mechanism, our previous study had indicated that Cx43 can interact with cell adhesion-associated proteins, such as E-cadherin, participating in the process of adhesion [21], which may propose a positive role of Cx43 expression in increasing cell adhesion.

The diapedesis of gastric cancer cells through mesothelial cell monolayers *in vitro* was further investigated in our experiments. Our study revealed that the diapedesis of Cx43-expressing gastric cells was significantly enhanced compared with Cx43T154A-expressing cells, suggesting that Cx43 expression affected cellular motility, which was consistent with several previous reports [2, 14, 22, and 23]. However, it remains to be elucidated whether Cx43 expression was associated with intercellular communication. We then explored whether GJIC plays a pivotal role in diapedesis. First, we examined the GJIC between gastric cancer cells and peritoneal mesothelial cells. The results demonstrated that Cx43-expressing gastric cancer cells established an effective heterotypic GJIC with peritoneal mesothelial cells; however, Cx43T154A-expressing cells failed to establish GJIC, as was considered before [10].

Cx43- and Cx43T154A-expressing gastric cells were added to mesothelial cell monolayers and co-cultured overnight (not longer than 12 hours) to further confirm the role of GJIC in the transmigration of gastric cancer cells through the peritoneal mesothelial cell monolayers. Gastric cancer cell diapedesis was examined using laser scanning confocal microscopy. Our results revealed that Cx43-expressing gastric cancer cells exhibited more efficient migration through the mesothelial cell monolayers than Cx43T154A and empty vector group cells, whereas the diapedesis efficiency of tumor cells in latter two groups was not significantly different, suggesting that the heterocellular GJIC between tumor and mesothelial cells is necessary to enhance diapedesis. To understand the pivotal role of GJIC in the diapedesis of gastric cancer cells through the mesothelial cell layers, we looked to previous studies that have elucidated the mechanisms involved in the diapedesis of tumor cells through endothelial cell layers. These studies have indicated that tumor cells may send signals to the endothelium, such as inositol triphosphate (IP3), through gap junctions to regulate intracellular calcium levels. Elevated levels of intracellular calcium transiently phosphorylate the myosin light chain to increase endothelial permeability. Endothelial cells may also send signals to tumor cells through gap junctions to upregulate cellular motility proteins, such as Rho GTPases, protein kinase C, and phosphoinositide-3 kinase [24–22,7]. These results can help to elucidate the mechanism of how GJIC plays a pivotal role during diapedesis of gastric cancer cells.

Taking into account the results of this study and recent findings, we suggest that Cx43-mediated gap junctions play an active role in the peritoneal metastasis of gastric cancer cells. Our results support the hypothesis that heterocellular gap-junctional couplings between gastric cancer cells and the mesothelium regulates the transmigration of gastric cancer cells to the peritoneal mesothelial cell monolayers. Gastric cancer cells’ transmigration through the mesothelial barrier occurred via paracellular migration. These results further revealed the underlying mechanisms related to the peritoneal metastasis of gastric cancer cells, which may provide therapeutic advantages for the design of drugs or reagents that inhibit heterocellular GJIC between gastric cancer and mesothelial cells to reduce the metastatic spread of tumor cells.
